# Identification of plasma lipidomic biomarkers for prognostic stratification in advanced gastric cancer treated with PD-1 inhibitor plus chemotherapy

**DOI:** 10.3389/fimmu.2026.1714472

**Published:** 2026-02-09

**Authors:** Zhouwei Zhan, Xiaojie Wang, Muqun He, Shenghong Wei, Jiami Yu, Hanchen Zheng, Qiaoting Hu, Bijuan Chen, Sha Huang, Zengqing Guo

**Affiliations:** 1 Department of Medical Oncology, Clinical Oncology School of Fujian Medical University, Fujian Cancer Hospital, Fuzhou, Fujian, China; 2 Department of Gastric Surgical Oncology, Clinical Oncology School of Fujian Medical University, Fujian Cancer Hospital, Fuzhou, Fujian, China; 3 Department of Radiation Oncology, Clinical Oncology School of Fujian Medical University, Fujian Cancer Hospital, Fuzhou, Fujian, China

**Keywords:** advanced gastric cancer, immunotherapy, metabolomics, nomogram, prognostic biomarker, risk score

## Abstract

**Background:**

Immunotherapy combined with chemotherapy has improved outcomes in advanced gastric cancer (GC), but reliable biomarkers to predict clinical benefit remain limited. Metabolomics provides a comprehensive assessment of systemic metabolic changes and may yield prognostic indicators to guide treatment selection.

**Methods:**

We performed untargeted liquid chromatography–tandem mass spectrometry (LC-MS/MS) metabolomics on baseline plasma from 40 patients with advanced GC receiving first-line programmed cell death protein-1 (PD-1) inhibitor plus chemotherapy. Patients were stratified into long-term survivors (LTS) and short-term survivors (STS) based on median overall survival (OS). Differential metabolites were identified using multivariate statistics, followed by univariate Cox regression and least absolute shrinkage and selection operator (LASSO) analysis to construct a metabolite-based risk score. Prognostic performance was evaluated using Kaplan-Meier analysis, time-dependent receiver operating characteristic (ROC) curves, and multivariate Cox models. Comparisons with conventional clinical factors were conducted, and a prognostic nomogram was developed. Proportional-hazards assumptions were assessed with Schoenfeld residuals; discrimination was optimism-corrected using 1,000-bootstrap resampling; Harrell’s concordance index (C-index), time-dependent area under the curve (AUC), and bootstrap-corrected calibration curves were reported. Twelve-month decision-curve analysis (DCA) quantified net clinical benefit compared with clinicopathologic baselines.

**Results:**

A total of 4,298 metabolites were detected, including 830 Level 1 and 1,321 Level 2 identifications. Principal component analysis and orthogonal partial least squares discriminant analysis showed clear separation between LTS and STS groups. Differential analysis revealed metabolites enriched in bile acid, amino acid, and retinol metabolism pathways. Cox and LASSO analyses identified six independent prognostic metabolites. The resulting metabolite-based risk score significantly stratified OS and progression-free survival (*p* < 0.01) and demonstrated stable predictive accuracy over 6–24 months. Compared with age, sex, tumor grade, and programmed death-ligand 1 combined positive score (PD-L1 CPS), the risk score showed superior discrimination. A nomogram integrating risk score, grade, and PD-L1 CPS yielded accurate OS predictions with strong calibration and higher net benefit in DCA. Internal validation supports the robustness of findings within this single-center, 40-patient cohort.

**Conclusions:**

A plasma metabolite-based risk score derived from six biomarkers independently predicts survival in advanced GC treated with PD-1–based immunotherapy and offers a practical tool for individualized prognosis.

## Introduction

Gastric cancer (GC) is one of the most common malignancies worldwide, ranking fifth in incidence and fourth in cancer-related mortality, with the majority of patients diagnosed at an advanced stage (1–3). Standard chemotherapy has long been the backbone of treatment, but survival outcomes remain unsatisfactory, with median overall survival (OS) rarely exceeding 12 months in advanced cases (4). The introduction of immune checkpoint inhibitors (ICIs), particularly antibodies targeting programmed cell death protein-1 (PD-1) or programmed death ligand-1 (PD-L1), has transformed the therapeutic landscape. Large phase III clinical trials, such as CheckMate-649 and ATTRACTION-4, demonstrated that combining ICIs with chemotherapy could extend progression-free survival (PFS) and OS compared with chemotherapy alone (5, 6). However, despite these advances, only a subset of patients benefits significantly, and both primary and acquired resistance remain major obstacles (7, 8).

Recent research highlights the complexity of immune evasion mechanisms in GC, ranging from genetic alterations and tumor mutational burden (TMB) heterogeneity to immunosuppressive tumor microenvironment (TME) features (8, 9). Established biomarkers such as PD-L1 expression, microsatellite instability (MSI), and Epstein–Barr virus (EBV) positivity offer partial predictive value, but they fail to fully capture the biological heterogeneity of GC (10). Therefore, beyond PD-L1 CPS and clinicopathologic features, there is a clear unmet clinical need for robust, clinically actionable biomarkers to predict benefit from first-line PD-1 inhibitor plus chemotherapy in advanced GC. In this context, multi-omics approaches, particularly metabolomics, have gained attention for their ability to reflect the dynamic metabolic interplay between tumor cells and the host immune system (11). By profiling metabolites in plasma (or serum), or tumor tissue, metabolomics provides an integrative readout of cellular function, offering insights into mechanisms of immune responsiveness and resistance.

Emerging evidence suggests that specific metabolic signatures are associated with immune checkpoint inhibitor (ICI) efficacy in solid tumors, including GC. Studies have shown that alterations in amino acid metabolism, lipid utilization, and energy pathways can modulate T cell activity, influence the immunosuppressive milieu, and ultimately shape therapeutic outcomes (12, 13). In non-small cell lung cancer (NSCLC), for instance, baseline metabolomic profiling has identified predictive metabolites linked to immunotherapy response (13, 14), a principle increasingly extended to gastrointestinal malignancies. In GC, emerging evidence highlights alterations in lipid metabolism and systemic inflammatory metabolites as potential predictors of treatment response (15, 16). Integrating metabolomics into predictive models holds the potential not only to refine patient selection but also to uncover actionable pathways for combination therapies. However, metabolomics-based biomarkers that can prospectively guide selection for PD-1 inhibitor plus chemotherapy in GC remain insufficiently defined and under validated. Against this backdrop, we sought to address this gap by applying plasma lipidomic to patients with advanced GC receiving first-line PD-1 inhibitor plus chemotherapy, aiming to develop and internally validate a metabolite-based risk model for survival stratification and to benchmark its performance against conventional clinical markers.

## Methods

### Study design and participants

This prospective observational study enrolled 40 patients with histologically confirmed advanced GC at Fujian Cancer Hospital between October 2022 and August 2024. Eligible patients had advanced GC and had not received prior systemic therapy. All participants received first-line PD-1 inhibitor combined with chemotherapy (oxaliplatin plus capecitabine) administered every 3 weeks until disease progression, unacceptable toxicity, or withdrawal. Baseline demographic and clinical data included age, sex, ECOG performance status (ECOG-PS), tumor differentiation grade, and PD-L1 combined positive score (CPS). Treatment efficacy was assessed according to RECIST v1.1 criteria. The primary endpoints were OS, defined as the time from treatment initiation to death from any cause or last follow-up, and PFS, defined as the time from treatment initiation to documented disease progression or death. Patients were followed regularly with imaging and clinical evaluation at predefined intervals until death or study completion in June 20, 2025. This study was approved by the Ethics Committee of Fujian Cancer Hospital, and all participants provided written informed consent.

### Sample collection and processing

Baseline peripheral blood samples were obtained from all patients prior to treatment initiation after overnight fasting. Approximately 5 mL of venous blood was collected in EDTA tubes, placed on ice, and processed within 2 h. Plasma was isolated by centrifugation at 3000 rpm for 10 min at 4°C, aliquoted to avoid repeated freeze–thaw cycles, and stored at −80°C until analysis. For metabolite extraction, plasma aliquots were mixed with pre-chilled methanol/acetonitrile solution containing internal standards, vortexed, sonicated on ice, and centrifuged at 12,000 rpm for 20 min at 4°C. The supernatant was transferred to autosampler vials for liquid chromatography-tandem mass spectrometry (LC-MS/MS) analysis. A pooled quality control (QC) sample, prepared by combining equal volumes of each plasma extract, was injected at regular intervals to monitor analytical stability (17, 18).

### LC-MS/MS profiling

Untargeted metabolomic profiling was performed using an ACQUITY UPLC I-Class Plus system (Waters, Milford, MA, USA) coupled to a Q Exactive Plus Orbitrap mass spectrometer (Thermo Fisher Scientific, Waltham, MA, USA) equipped with a heated electrospray ionization (HESI) source. Chromatographic separation was achieved on an HSS T3 column (100 × 2.1 mm, 1.8 μm) maintained at 45°C. The mobile phases consisted of (A) water with 0.1% formic acid and (B) acetonitrile with 0.1% formic acid, with a linear gradient elution from 5% to 100% B over 15 min at a flow rate of 0.35 mL/min. The injection volume was 5 μL, and the autosampler temperature was set to 10°C. The mass spectrometer was operated in both positive and negative ionization modes. Full MS scans were acquired over an m/z range of 70–1050 with a resolution of 60,000, followed by data-dependent MS/MS at a resolution of 15,000 using stepped normalized collision energies (10, 20, and 40 eV). Source parameters included a spray voltage of +3.8/−3.2 kV, capillary temperature of 320°C, sheath gas at 35 arb, and auxiliary gas at 8 arb. QC samples were injected every 10 samples to monitor retention time, mass accuracy, and signal intensity stability throughout the analytical sequence (18, 19).

### Data processing and identification

Raw LC-MS/MS data were processed using Progenesis QI (version 2.3, Nonlinear Dynamics, UK) for peak detection, retention time alignment, deconvolution, and normalization (precursor tolerance 5 ppm; fragment tolerance 10 ppm; product-ion threshold 5%). To control instrumental drift, features were corrected by a pooled-QC-guided locally estimated scatterplot smoothing (LOESS) procedure, also known as QC-based robust LOESS signal correction (QC-RLSC; degree = 2, span = 0.75), applied per ion mode and re-centered to the QC median; no class labels were used at any step. Features with missing values in more than 50% of samples per group were excluded; remaining missing values were handled by a tiered strategy: zeros consistent with limits-of-detection were imputed at one-half of the minimum non-zero intensity within ion-mode/batch, whereas sporadic missing completely at random (MCAR)-like gaps were imputed by k-nearest neighbors (k = 5) after Pareto scaling. Data were log_2_-transformed prior to analysis. Metabolite annotation followed the Metabolomics Standards Initiative (MSI) guidelines (20). Identification was performed by matching accurate mass, isotope distribution, and MS/MS spectra against multiple databases, including the Human Metabolome Database (HMDB), LipidMaps (v2.3), METLIN, and the LuMet-Animal 3.0 in-house spectral library. Putative matches required an identification score ≥36 (out of 80) using the Progenesis QI (v2.3) identification score, which integrates four components—accurate mass error, MS/MS fragment-similarity, isotope-pattern fit, and retention-time (RT) agreement—each weighted up to 20 points (21, 22). Identified metabolites were classified into four confidence levels: Level 1, confirmed by analysis of authentic reference standards under identical LC–MS conditions with concordant RT (± 0.3 min) and high-quality mirror-plot MS/MS spectra (in addition to accurate mass and isotope fit); Level 2, putatively annotated compounds based on library/reference MS/MS and accurate mass/isotope evidence without co-analyzed standards/RT confirmation; Level 3, putatively characterized compound classes; and Level 4, unknowns. For downstream analyses, only Level 1–2 metabolites were retained and further grouped into superclass, class, and subclass categories according to HMDB and LipidMaps ontologies.

### Statistics and modeling

Data preprocessing and statistical analyses were performed using R software. Data were Pareto-scaled prior to multivariate analysis. Principal component analysis (PCA) was used to assess data quality and clustering, while partial least squares-discriminant analysis (PLS-DA) and orthogonal PLS-DA (OPLS-DA) were applied to distinguish between the long-term survivor (LTS) and short-term survivor (STS) groups. Model validity for OPLS-DA was evaluated with 200 label permutations (no class-label leakage). Differential metabolites were identified based on variable importance in projection (VIP) > 1.0 plus Student’s t-test *p* < 0.05, with fold change (FC) thresholds of ≥1.2 or ≤0.83. The Benjamini-Hochberg false discovery rate (FDR) was applied to adjust for multiple testing. For visualization and descriptive comparisons only, LTS/STS grouping was derived *post hoc* by the median OS; all inferential survival modeling used uncategorized time-to-event data. For survival analysis, only MSI Level 1–2 lipid and lipid-like metabolites were considered. Univariate Cox regression was used to screen OS-associated metabolites (*p* < 0.01), followed by least absolute shrinkage and selection operator (LASSO) regression to reduce dimensionality and prevent overfitting. The LASSO penalty parameter (λ) was tuned by 10-fold cross-validation (glmnet), and the final model was selected at λ_min (minimum cross-validated partial-likelihood deviance). A multivariate Cox proportional hazards model was then constructed to generate a metabolic risk score. Model performance was evaluated by Kaplan-Meier survival analysis, time-dependent receiver operating characteristic (ROC) curves, and nomogram calibration.

### Internal validation, calibration, and decision curve analysis

To address model optimism in the cohort of 40 patients, we applied bootstrap optimism correction using 1,000 resamples (Harrell’s method) and reported bias-adjusted discrimination, including the C-index and time-dependent area under the curve (AUC) at 6, 12, 18, and 24 months with 95 percent confidence intervals. Time-dependent AUCs were computed using the riskRegression Score function, and percentile bootstrap was used for the C-index. Proportional hazards assumptions were tested using Schoenfeld residuals (cox.zph) and further verified by visual inspection. Calibration was evaluated with the rms calibrate function using 1,000 bootstrap resamples to obtain optimism-corrected calibration curves and confidence bands at 6, 12, and 18 months. Clinical utility was assessed through decision curve analysis at 12 months using the rmda package, comparing the risk score with age, sex, grade, and programmed death ligand 1 combined positive score. Given the single-center design and internal validation, performance measures are interpreted as hypothesis-generating and require external validation. All analyses were conducted in R version 4.4.1 (R Foundation for Statistical Computing, Vienna, Austria) using the survival 3.8-3, glmnet 4.1-10, timeROC 0.4, riskRegression 2025.09.17, rms 8.0-0, survminer 0.5.1, rmda 1.6, ggplot2 4.0.0, ComplexHeatmap 2.24.1, and circlize 0.4.16 packages.

## Results

### Baseline characteristics and survival outcomes

**Figure 1:**
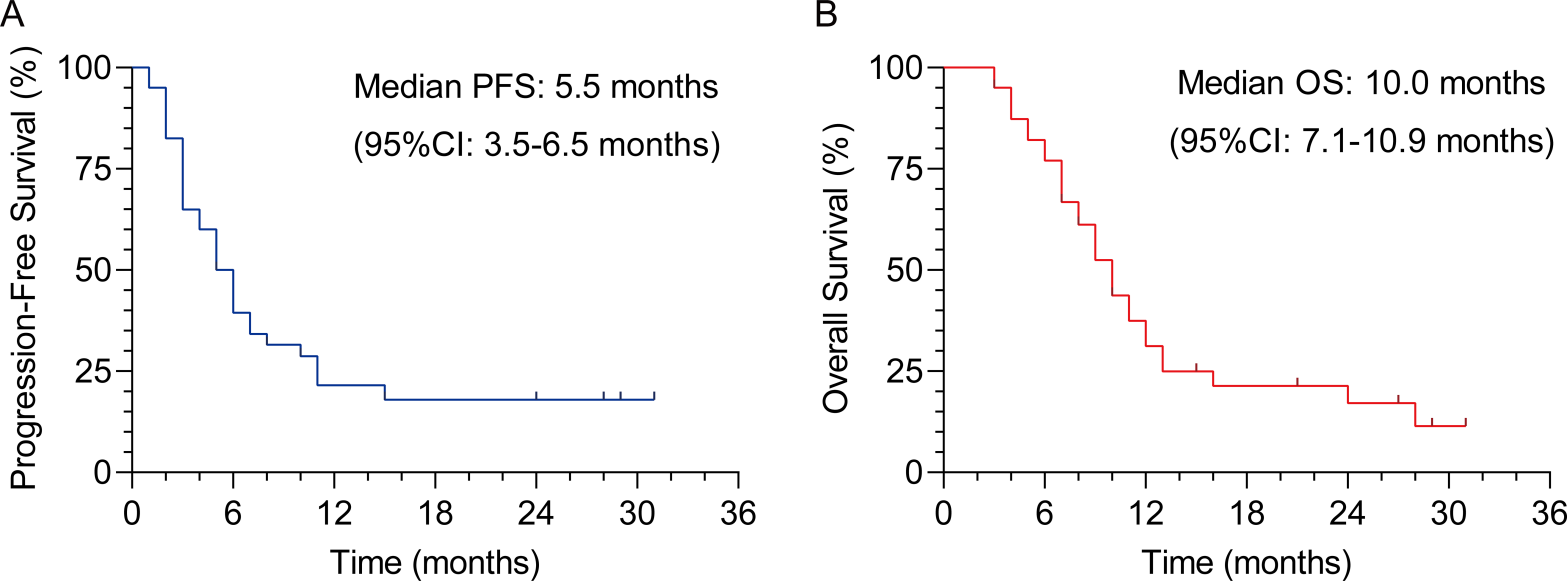
Kaplan-Meier survival analysis of patients with advanced gastric cancer (N = 40). **(A)** Progression-free survival (PFS) curve of the study cohort, displayed with 95% confidence intervals and log-rank test *p* value. **(B)** Overall survival (OS) curve of the study cohort, displayed with 95% confidence intervals and log-rank test *p* value.

As shown in **Table 1**, the study cohort consisted of 40 patients with advanced GC, with a slight predominance of males and a majority aged over 60 years. ECOG performance status was generally favorable, with most patients classified as 0, and the majority of tumors exhibited poor differentiation. When stratified into LTS and STS according to median OS, the distribution of age, sex, ECOG score, tumor differentiation, and PD-L1 CPS showed no significant differences between groups. During a median follow-up of 27.0 months (interquartile range, 19.3-34.7), OS events occurred in 30 of 40 patients (75.0%), and PFS events occurred in 31 of 40 patients (77.5%). Kaplan-Meier survival analysis (**Figure 1**) indicated a median PFS of 5.5 months (95% CI: 3.5-6.5) and a median OS of 10.0 months (95% CI: 7.1-10.9) in the entire cohort. These data illustrate relatively uniform baseline features across subgroups and the OS patterns of patients receiving PD-1 inhibitor combined with chemotherapy.

**Table 1:** Baseline characteristics of patients.

Characteristic	Total (n=40)	LTS (n=20)	STS (n=20)	*P* value
Gender, n (%)				0.197
Male	24 (60.0%)	14 (70.0%)	10 (50.0%)	
Female	16 (40.0%)	6 (30.0%)	10 (50.0%)	
Age (years), n (%)				0.057
< 60	18 (45.0%)	6 (30.0%)	12 (60.0%)	
≥ 60	22 (55.0%)	14 (70.0%)	8 (40.0%)	
ECOG PS, n (%)				0.507
0	26 (65.0%)	14 (70.0%)	12 (60.0%)	
1	14 (35.0%)	6 (30.0%)	8 (40.0%)	
Tumor differentiation, n (%)				0.311
High-Moderate	13 (32.5%)	8 (40.0%)	5 (25.0%)	
Poor	27 (67.5%)	12 (60.0%)	15 (75.0%)	
PD-L1 expression				0.110
PD-L1 positive (CPS ≥ 5)	11 (27.5%)	6 (30.0%)	5 (25.0%)	
PD-L1 negative (CPS < 5)	29 (72.5%)	14 (70.0%)	15 (75.0%)	

LTS, Long-term Survivors; STS, Short-term Survivors.

### Metabolomic detection and QC results

A total of 4,298 metabolic features were detected in plasma samples after data preprocessing. QC assessment confirmed the robustness of the analytical platform. PCA showed tight clustering of QC samples, indicating consistent performance across runs. Pairwise correlation coefficients among QC samples exceeded 0.99, and over 80% of detected metabolites exhibited a relative standard deviation (RSD) below 30%. After filtering out peaks with more than 50% missing values per group and replacing zero values with half-minimum intensities, the positive- and negative-ion data were merged into a unified dataset for downstream analyses (**Supplementary Figure 1**). According to the Metabolomics Standards Initiative classification, 830 metabolites were annotated as Level 1, 1,321 as Level 2, 564 as Level 3, and 1,583 as Level 4. Only metabolites annotated at MSI Level 1 or Level 2 were retained for all downstream analyses. Lipid-related metabolites predominated across categories, with glycerophospholipids, fatty acyls, and carboxylic acid derivatives accounting for the largest proportions at the Class and Sub Class levels.

### Multivariate statistical analysis and identification of differential metabolites

**Figure 2:**
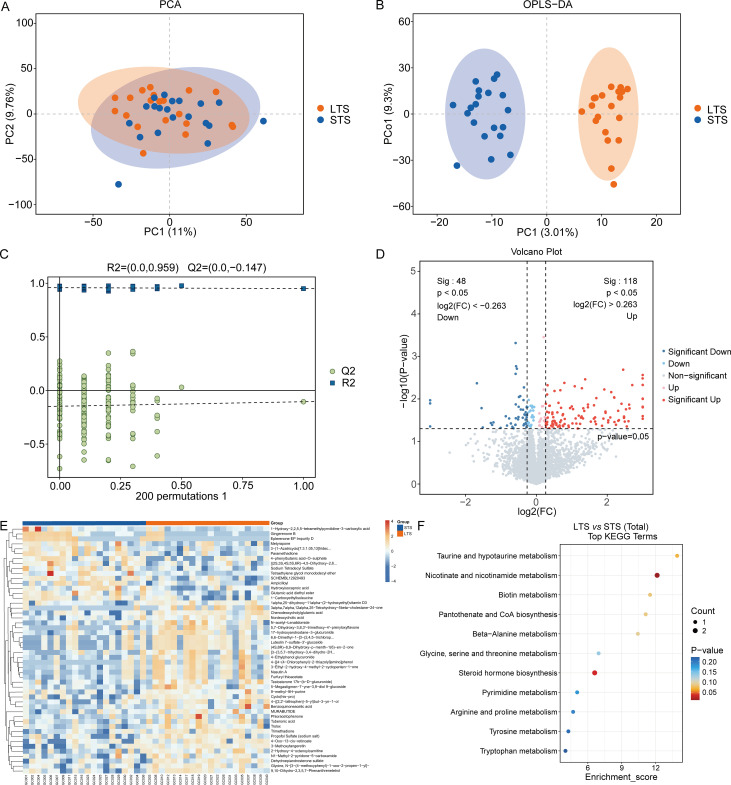
Multivariate statistical analysis and identification of differential metabolites between groups. Groups: LTS n = 20, STS n = 20 (total N = 40). **(A)** Principal component analysis (PCA) score plot displaying the global distribution of metabolic profiles between STS and LTS groups. **(B)** Orthogonal partial least squares discriminant analysis (OPLS-DA) score plot showing clearer group separation. **(C)** Permutation test with 200 permutations confirming the validity and reliability of the OPLS-DA model. **(D)** Volcano plot of differential metabolites; red and blue dots represent significantly upregulated and downregulated metabolites (*p* < 0.05, two-sided Student’s t-tests with Benjamini-Hochberg false discovery rate (BH-FDR) correction; FC thresholds ≥1.2 or ≤0.83). **(E)** Heatmap of significantly altered metabolites, visualizing clustering patterns between groups. **(F)** KEGG pathway enrichment analysis of differential metabolites, highlighting the top 10 significantly enriched metabolic pathways (FDR < 0.05; N = 166 metabolites entered).

PCA revealed clear separation trends between the LTS and STS groups, reflecting distinct metabolic profiles (**Figure 2A**). OPLS-DA further improved group discrimination (**Figure 2B**), and 200 permutations confirmed the robustness and lack of overfitting of the OPLS-DA model (**Figure 2C**). Differential metabolite screening identified a large set of significantly altered metabolites between the LTS and STS groups. The volcano plot illustrated both upregulated and downregulated features (*p* < 0.05) (**Figure 2D**). Hierarchical clustering analysis of these metabolites showed clear group-specific patterns (**Figure 2E**). Kyoto Encyclopedia of Genes and Genomes (KEGG) enrichment analysis revealed significant involvement of multiple pathways, with the top enriched categories including bile acid metabolism, amino acid metabolism, and retinol metabolism (**Figure 2F**).

### Identification of prognostic metabolites and risk score development

**Figure 3:**
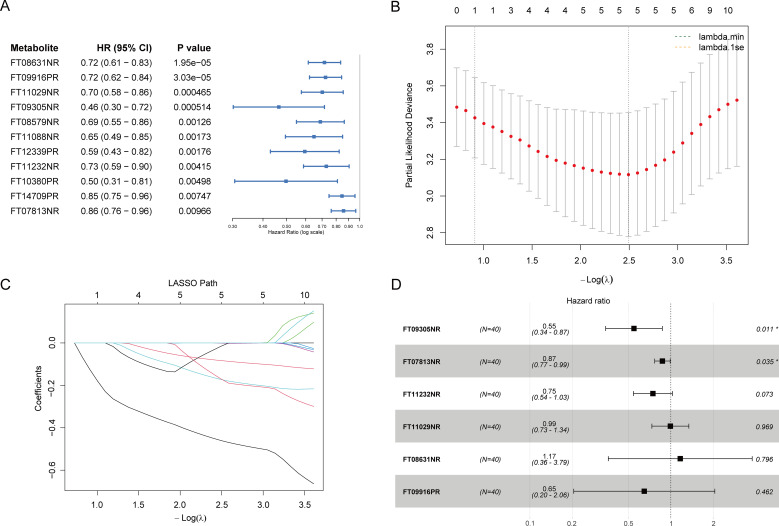
Identification of prognostic metabolites by univariate Cox regression and LASSO analysis. Analytic set: N = 40; outcomes based on OS events = 30. **(A)** Forest plot of univariate Cox proportional hazards regression showing HRs and 95% CIs for 11 candidate metabolites significantly associated with OS (*p* < 0.01). The metabolites include FT08631NR (deoxycholic acid), FT09916PR (cholenic acid), FT11029NR (3’-hydroxy-T2 Toxin), FT09305NR (5-megastigmen-7-yne-3,9-diol 9-glucoside), FT08579NR (12α-hydroxy-7-oxo-5α-cholan-24-oic acid), FT11088NR (17-hydroxyandrostane-3-glucuronide), FT12339PR (lucidenic acid C), FT11232NR (corticosterone-21-hemisuccinate), FT10380PR (5,6β-Dihydro PGI2), FT14709PR (colocynthenin F), and FT07813NR (4-oxo-13-cis-retinoate). **(B)** Ten-fold cross-validation curve for LASSO-Cox; vertical line indicates λ_min (final model); the dashed line marks λ_1se for reference. **(C)** LASSO coefficient profiles of the 11 metabolites with varying log(λ) values. **(D)** Multivariate Cox regression model based on LASSO-selected metabolites showing HRs and 95% CIs, with global model *p* value, Akaike information criterion (AIC), and concordance index (C-index) reported.

From the pool of differential metabolites, Level 1 and Level 2 lipids and lipid-like molecules (n = 33) were prioritized for survival analysis. Univariate Cox regression identified 11 metabolites significantly associated with OS (*p* < 0.01), including deoxycholic acid (FT08631NR), cholenic acid (FT09916PR), 3’-hydroxy-T2 toxin (FT11029NR), 5-megastigmen-7-yne-3,9-diol 9-glucoside (FT09305NR), 12α-hydroxy-7-oxo-5α-cholan-24-oic acid (FT08579NR), 17-hydroxyandrostane-3-glucuronide (FT11088NR), lucidenic acid C (FT12339PR), corticosterone-21-hemisuccinate (FT11232NR), 5,6β-dihydro PGI2 (FT10380PR), colocynthenin F (FT14709PR), and 4-oxo-13-cis-retinoate (FT07813NR) (**Figure 3A**). LASSO regression with ten-fold cross-validation was then applied to refine the candidate set, yielding an optimal λ value that minimized partial likelihood deviance (**Figure 3B**). The coefficient profiles across varying λ values demonstrated progressive shrinkage of variables (**Figure 3C**). Subsequent multivariate Cox regression ultimately identified six independent prognostic metabolites: deoxycholic acid (FT08631NR), cholenic acid (FT09916PR), 3’-hydroxy-T2 toxin (FT11029NR), 5-megastigmen-7-yne-3,9-diol 9-glucoside (FT09305NR), corticosterone-21-hemisuccinate (FT11232NR), and 4-oxo-13-cis-retinoate (FT07813NR). These metabolites were integrated into a metabolite-based risk score, which showed independent prognostic value with hazard ratios (HRs), 95% CIs (**Figure 3D**).

### Prognostic value of the metabolite-based score for OS and PFS

**Figure 4:**
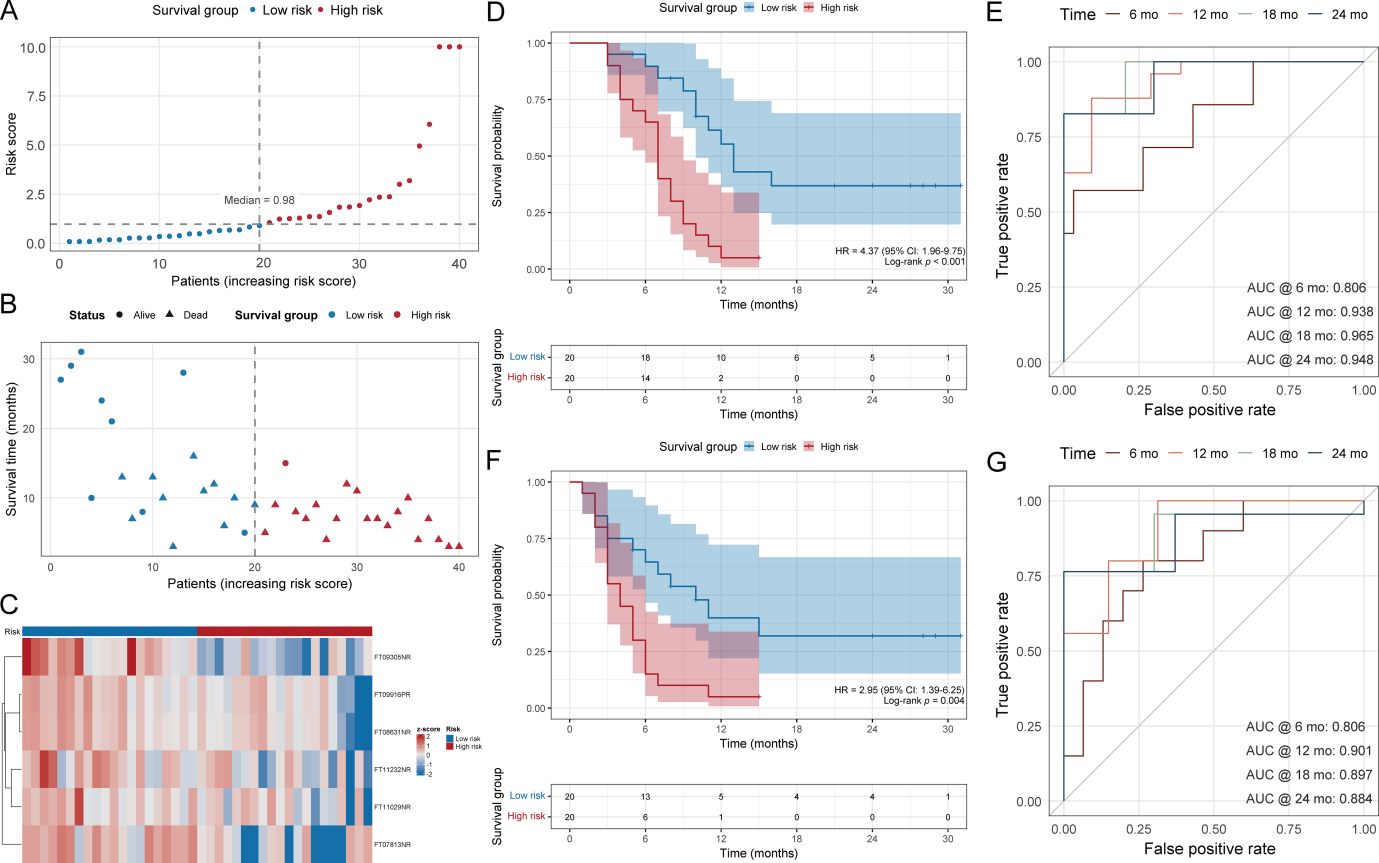
Prognostic significance of the metabolite-based risk score for OS and PFS. Analytic set: N = 40; outcomes based on OS events = 30. **(A)** Distribution of patients according to the risk score, with the median value (0.98) as the cutoff defining low- and high-risk groups. **(B)** Scatter plot of OS aligned with the risk score; circles indicate censored patients and triangles indicate deaths. **(C)** Heatmap of six model metabolites [FT09305NR (5-megastigmen-7-yne-3,9-diol 9-glucoside), FT09916PR (cholenic acid), FT08631NR (deoxycholic acid), FT11232NR (corticosterone-21-hemisuccinate), FT11029NR (3’-hydroxy-T2 Toxin), and FT07813NR (4-oxo-13-cis-retinoate)] showing differential expression between low- and high-risk groups. **(D)** Kaplan-Meier curves of OS stratified by the risk score, with HR, 95% CI, and log-rank test results indicated. **(E)** Time-dependent ROC curves evaluating predictive performance of the risk score for OS at 6, 12, 18, and 24 months, with corresponding AUC values and 95% confidence interval ribbons estimated by bootstrap resampling. **(F)** Kaplan-Meier curves of PFS stratified by the risk score, with HR, 95% CI, and log-rank test results indicated. **(G)** Time-dependent ROC curves evaluating predictive performance of the risk score for PFS at 6, 12, 18, and 24 months, with corresponding AUC values and 95% confidence interval ribbons estimated by bootstrap resampling.

The metabolite-based risk score, derived from six independent prognostic metabolites, was applied to stratify patients into LTS and STS groups using the median score (0.98) as cutoff. Distribution plots showed that higher risk scores were predominantly enriched in the STS group (**Figure 4A**). Scatter plots aligned with clinical outcomes demonstrated that patients with elevated scores experienced earlier events, while lower scores were associated with prolonged OS (**Figure 4B**). Heatmap analysis of the six model metabolites [FT09305NR (5-megastigmen-7-yne-3,9-diol 9-glucoside), FT09916PR (cholenic acid), FT08631NR (deoxycholic acid), FT11232NR (corticosterone-21-hemisuccinate), FT11029NR (3’-hydroxy-T2 Toxin), and FT07813NR (4-oxo-13-cis-retinoate)] confirmed distinct expression patterns between LTS and STS patients (**Figure 4C**). Kaplan-Meier analyses showed that patients with high scores had significantly shorter OS and PFS compared with those in the low-score group (**Figures 4D, F**). The predictive accuracy of the risk score was further validated by time-dependent ROC curves, which demonstrated robust performance with consistently high AUC values at 6, 12, 18, and 24 months for both OS and PFS (**Figures 4E, G**).

### Integrated comparison with clinical factors and nomogram construction

**Figure 5:**
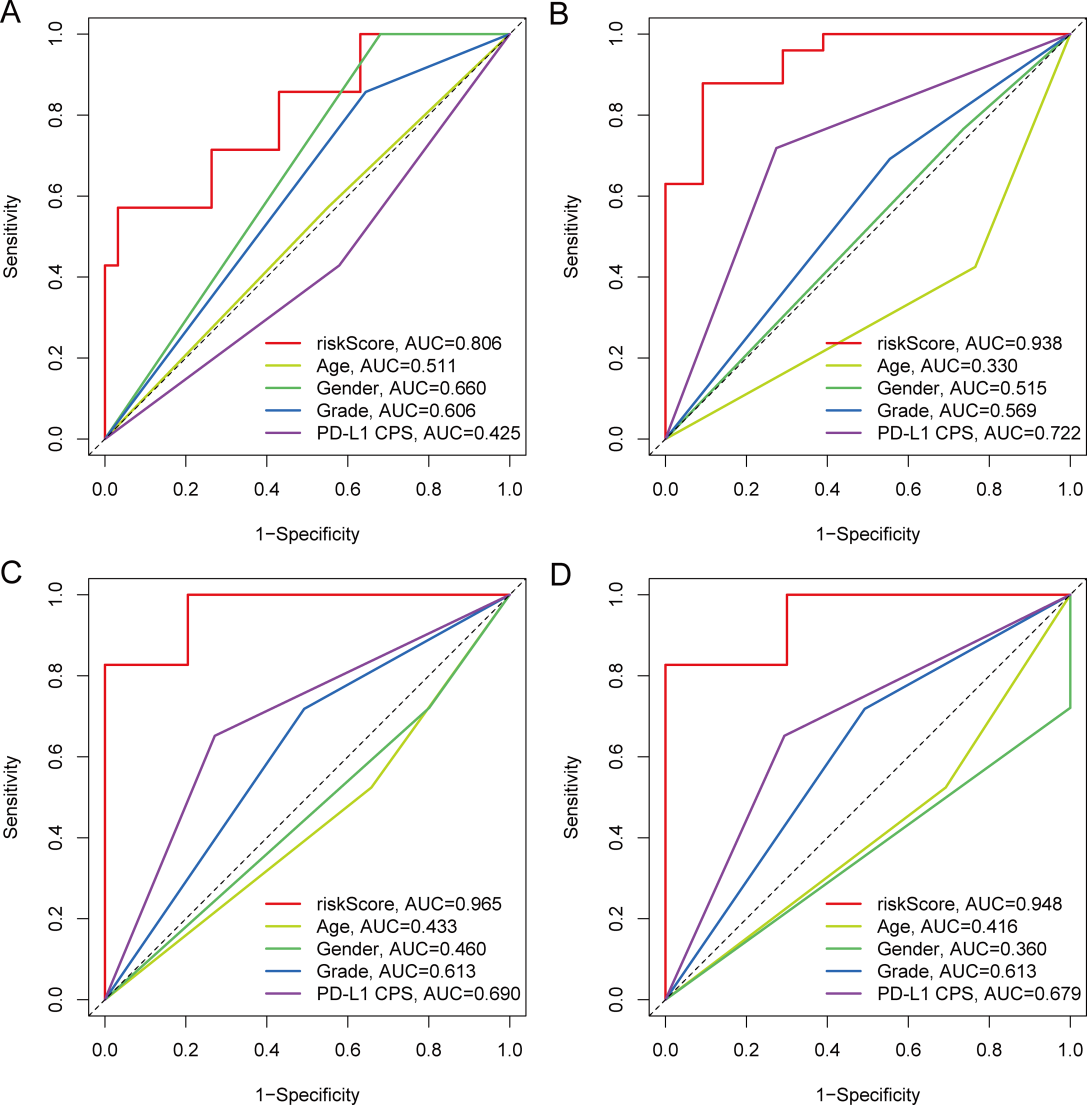
Comparison of predictive performance between the metabolite-based risk score and clinical parameters. Cohort size: N = 40. Evaluation times: 6, 12, 18, and 24 months. **(A–D)** Time-dependent ROC curves comparing the predictive ability of the metabolite-based risk score with conventional clinical factors, including age, gender, tumor differentiation grade, and PD-L1 CPS, at 6 months **(A)**, 12 months **(B)**, 18 months **(C)**, and 24 months **(D)**. The AUC values are indicated for each factor, with 95% confidence interval ribbons derived from bootstrap resampling.

**Figure 6:**
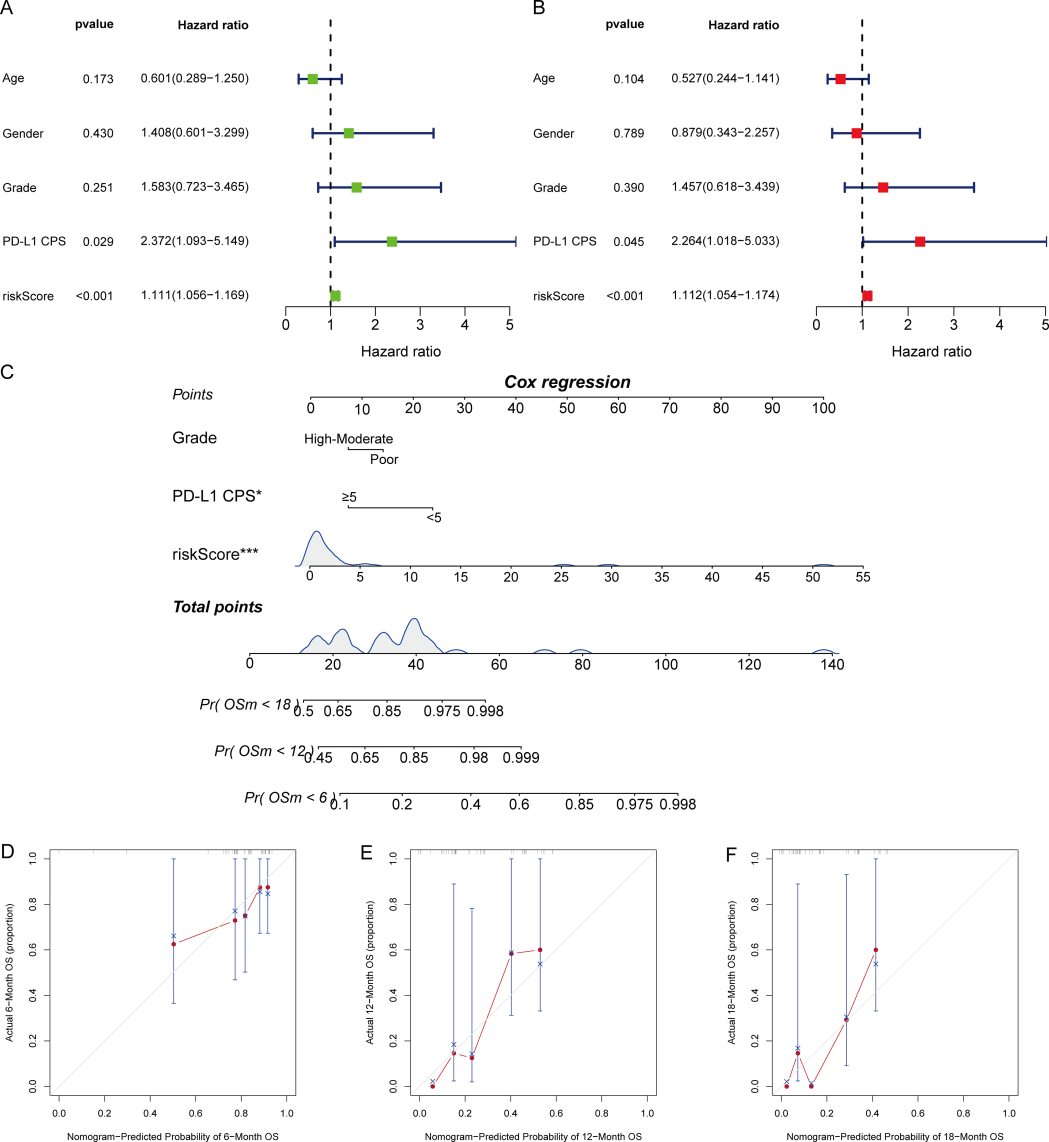
Construction and validation of the nomogram model integrating the metabolite-based risk score. Cohort size: N = 40; outcomes based on OS events = 30. **(A)** Univariate Cox regression of clinical variables and metabolite-based risk score for OS. **(B)** Multivariate Cox regression analysis confirming the independent prognostic value of the risk score. **(C)** Nomogram integrating risk score, tumor differentiation grade, and PD-L1 CPS to predict 6-, 12-, and 18-month OS probabilities. **(D–F)** Bootstrap-corrected calibration plots comparing predicted versus observed survival at 6 **(D)**, 12 **(E)**, and 18 months **(F)**, displayed as calibration curves with 95% confidence interval ribbons, showing good agreement.

To further assess the prognostic value of the metabolite-based risk score, its predictive performance was compared with conventional clinical variables, including age, gender, tumor grade, and PD-L1 CPS. Time-dependent ROC analyses demonstrated that the risk score consistently outperformed these clinical parameters at 6, 12, 18, and 24 months, with higher AUC values across all time points (**Figures 5A–D**). Univariate Cox regression analysis indicated that the risk score, tumor grade, and PD-L1 CPS were associated with OS (**Figure 6A**). Multivariate analysis confirmed the risk score as an independent prognostic factor (**Figure 6B**). A nomogram integrating the metabolite-based risk score with grade and PD-L1 CPS was then developed to predict 6-, 12-, and 18-month OS probabilities (**Figure 6C**). Calibration plots showed good agreement between predicted and OS outcomes, supporting the robustness of the integrated model (**Figures 6D–F**).

### Model diagnostics, optimism-correction, and clinical utility

Cox proportional-hazards assumptions were not violated for the metabolite-based risk score or covariates based on global and variable-level Schoenfeld tests, with diagnostic plots showing no systematic time trends (**Supplementary Figure S2**). Clinical utility at 12 months favored the metabolite risk score: DCA demonstrated higher net benefit across a wide range of threshold probabilities versus “treat-all,” “treat-none,” and clinicopathologic baselines (age, sex, grade, PD-L1 CPS) (**Supplementary Figure S3**). To address optimism in the n = 40 cohort, we performed 1,000-bootstrap optimism correction. The bias-adjusted Harrell’s C-index and time-dependent AUCs at 6/12/18/24 months are reported with 95% confidence intervals in **Supplementary Table S1**; overall discrimination remained moderate-to-high after correction, and optimism-corrected calibration showed close agreement between predicted and observed survival with narrow confidence bands at prespecified time points.

## Discussion

We profiled baseline plasma metabolomes in advanced GC patients receiving first-line PD-1 inhibitor plus chemotherapy and identified lipid-derived metabolites as prognostic biomarkers. A six-metabolite risk score, composed of deoxycholic acid, cholenic acid, 3′-hydroxy-T2 toxin, 5-megastigmen-7-yne-3,9-diol 9-glucoside, corticosterone-21-hemisuccinate, and 4-oxo-13-cis-retinoate, significantly stratified both OS and PFS. The score outperformed clinicopathological factors (age, gender, grade, PD-L1 CPS), highlighting added value beyond standard markers. Time-dependent ROC analyses showed stable discrimination at early (6–12 months) and late (18–24 months) landmarks, supporting both early triage of potential non-responders and longer-horizon prognosis assessment. Proportional-hazards assumptions were satisfied. Using 1,000-bootstrap optimism correction, bias-adjusted Harrell’s C-index and time-AUCs with 95% confidence intervals remained consistent with apparent estimates, supporting robustness despite the modest sample. Bootstrap-corrected calibration curves with confidence bands demonstrated close agreement between predicted and observed survival. DCA at 12 months showed higher net clinical benefit for the metabolite score than clinicopathologic baselines across clinically relevant threshold probabilities. Given the single-center, n=40 cohort and reliance on internal validation, we present the work as hypothesis-generating. Overall, lipidomics offers a complementary, clinically informative layer for survival prediction and treatment stratification in GC immunotherapy, warranting external validation and prospective evaluation for clinical translation.

Previous studies have increasingly emphasized the role of metabolic reprogramming in modulating cancer progression and shaping responses to immunotherapy. Several metabolomic investigations in lung cancer and hepatocellular carcinoma have reported that alterations in lipid metabolism strongly correlate with checkpoint inhibitor efficacy (23–25). In NSCLC, for example, branched-chain amino acid and bile acid pathways were identified as predictive markers of therapeutic outcomes (26), while in hepatocellular carcinoma, combined proteomic and metabolomic profiling revealed distinct lipid signatures linked to favorable responses to PD-1 inhibitors (27). Our findings are consistent with these reports, as we identified six lipid-associated metabolites that stratified patients according to survival outcomes. Moreover, recent studies have highlighted bile acid derivatives such as deoxycholic acid as regulators of immune signaling within the tumor microenvironment (28, 29), supporting the relevance of our observations. However, evidence specific to GC remains limited, and our study contributes novel insights by demonstrating that lipid metabolites, rather than conventional clinical biomarkers, provide stronger prognostic value for immunotherapy outcomes in this patient population.

The six lipid-related metabolites identified in this study may influence immunotherapy outcomes through distinct biological pathways. Deoxycholic acid and cholenic acid, both bile acid derivatives, are known modulators of gut microbiota and immune regulation, potentially affecting antigen presentation and T-cell activation (30, 31). Elevated bile acids have been shown to shape the tumor immune microenvironment by altering dendritic cell function and promoting immunosuppressive phenotypes. 3’-hydroxy-T2 toxin, a trichothecene metabolite, may impact immune function via oxidative stress and modulation of cytokine release, potentially influencing tumor-associated inflammation (32). 5-megastigmen-7-yne-3,9-diol 9-glucoside, a carotenoid derivative, could exert immunomodulatory effects through antioxidant activity and retinoid-related signaling, which are implicated in T-cell differentiation (33, 34). Corticosterone-21-hemisuccinate, a glucocorticoid analog, may directly influence immune checkpoints by modulating T-cell proliferation and PD-1/PD-L1 axis activity, thereby affecting the efficacy of checkpoint inhibitors (35). Finally, 4-oxo-13-cis-retinoate, a retinoic acid metabolite, is strongly linked to retinoid signaling, which has been shown to enhance cytotoxic T-cell function and synergize with PD-1 blockade in preclinical models (36, 37). Collectively, these metabolites converge on pathways involving bile acid metabolism, retinoid signaling, and steroid hormone regulation, which are increasingly recognized as modulators of tumor-immune interactions. To our knowledge, this is the first report linking this specific six-metabolite plasma lipid signature to survival in a gastric cancer cohort treated with first-line PD-1 inhibitor plus chemotherapy; accordingly, these associations should be considered hypothesis-generating. Two metabolites are MSI Level-2 (*putative*) annotations, and the exact mechanistic roles of all six within the gastric cancer immune microenvironment require orthogonal experimental validation (e.g., standard-confirmed LC–MS/MS, functional assays, and longitudinal sampling).

From a clinical and policy perspective, the integration of metabolomics-derived biomarkers into GC management has potential to transform patient care. Currently, treatment decisions for advanced GC largely rely on clinicopathologic features and PD-L1 expression, yet these factors provide limited predictive value for immunotherapy response (38). In our cohort, PD-L1 CPS showed modest discrimination, which may relate to small single-center sample size, a CPS distribution skewed toward low–intermediate values, assay/scoring variability (immunohistochemistry clone, fixation, interobserver effects), spatial–temporal tumor heterogeneity relative to plasma sampling, and attenuation of PD-L1’s gradient in the chemo-immunotherapy setting; the binary CPS ≥5 vs <5 threshold may also compress signal. Our study suggests that lipid-based metabolite signatures can serve as complementary tools to improve prognostic accuracy, thereby informing individualized treatment strategies. Importantly, the risk score exhibited temporally stable performance at early (e.g., 6 months) and late (e.g., 24 months) landmarks, supporting both early triage of potential non-responders (to consider regimen adjustment or trial referral) and longer-horizon prognosis planning. For example, patients with unfavorable metabolic profiles could be considered for intensified treatment regimens or close monitoring, while those with favorable signatures might safely continue immunotherapy. On a broader scale, such biomarkers could support precision oncology frameworks and guide resource allocation by identifying subgroups most likely to benefit from costly immunotherapies. To enable clinical translation, implementation will require standardized pre-analytics/analytics (SOPs for plasma handling, LC–MS settings, and identification criteria), external validation across centers, and health-economic evaluation of turnaround time and cost. Furthermore, incorporating metabolomics into clinical trials could refine patient selection and accelerate the development of tailored therapeutic combinations, advancing the paradigm of biomarker-driven oncology (39).

This study has several strengths. It is among the first to systematically evaluate lipid-based metabolomic signatures in advanced GC patients receiving immunotherapy, leveraging comprehensive LC-MS/MS profiling combined with rigorous statistical modeling to derive robust prognostic markers. The integration of multivariate analyses and validation across both OS and PFS endpoints enhances the credibility of the findings. Nonetheless, some limitations should be acknowledged. The sample size, though adequate for exploratory modeling, may limit the generalizability of results, and external validation in larger, independent cohorts is required. Two model features are MSI Level-2 (*putative*) identifications without same-run standards, and the LTS/STS split was used only for visualization, not inference. Furthermore, while we identified six key lipid metabolites associated with survival, their mechanistic roles in immune modulation remain incompletely understood. In addition, the pathway-level interpretation of these metabolites is based on biochemical annotation and prior literature rather than direct multi-omic validation; we did not formally correlate pathway activity with immune-cell infiltration or immune-response signatures in public gastric cancer datasets, and these links should therefore be regarded as hypothesis-generating. Next steps will include (i) prospective, multicenter validation with harmonized SOPs and prespecified metrics (discrimination, calibration, DCA), (ii) targeted LC–MS/MS confirmation of key metabolites using authentic standards to enable a deployable assay, and (iii) development of a multimodal model integrating the metabolite risk score with radiomics (contrast-enhanced CT features) and genomic/immune markers (e.g., TMB/MSI status, PD-L1 CPS, RNA immune signatures). Future studies should integrate multi-omics approaches, including transcriptomics and microbiome profiling, to elucidate the biological pathways linking lipid metabolism and immunotherapy outcomes. Future studies should incorporate multi-omics strategies, particularly by combining plasma metabolomics, tumor transcriptomics, and computational immune profiling, to clarify the biological pathways that connect lipid metabolism, immune cell infiltration, and responses to immunotherapy. Prospective trials incorporating metabolomic monitoring are also warranted to confirm clinical utility and facilitate translation into routine practice. A health-economic assessment of turnaround time, cost, and workflow feasibility will further inform implementation.

## Conclusions

This study underscores the potential of metabolomics to provide novel insights into the prognosis and treatment stratification of advanced GC patients receiving immunotherapy. By integrating lipid-related metabolic markers with clinical outcomes, the work highlights the value of metabolite-based models as tools to refine patient selection and improve precision oncology approaches. Beyond prognostic significance, the findings emphasize the broader importance of metabolic pathways in shaping the tumor-immune interface, supporting the concept that systemic metabolism and immune regulation are closely intertwined. These results suggest that future research should expand toward multi-omics integration, combining metabolomics with proteomics, transcriptomics, and microbiome profiling to uncover robust biomarkers and mechanistic drivers of treatment response. We plan a prospective, multicenter validation to test transportability across institutions and platforms, alongside targeted LC–MS/MS standard-confirmed assays for key metabolites. We will also explore a multimodal predictive framework by combining the metabolite risk score with radiomics and genomic/immune features to enhance clinical utility, supported by prespecified performance and clinical-utility analyses. Additionally, validation in larger multicenter cohorts and functional studies are warranted to translate these discoveries into clinically applicable strategies. Health-economic evaluation and workflow optimization will guide real-world deployment. Ultimately, such approaches may pave the way for metabolically informed immunotherapy and more personalized therapeutic decision-making in GC.

## Data Availability

The raw liquid LC/MS/MS data generated in this study have been deposited in the OMIX repository of the National Genomics Data Center (NGDC)/China National Center for Bioinformation (CNCB) under accession number OMIX012655 and are available at https://ngdc.cncb.ac.cn/omix. All other data supporting the findings of this study are included in the article and its **Supplementary Materials** or can be obtained from the corresponding authors upon reasonable request.

## Supplementary material

Supplementary Figure 1 QC assessment and global structure of the lipidomics dataset. **(A)** PCA score plot showing overall dispersion of pooled-QC (green), short-term survivors (STS, blue), and long-term survivors (LTS, orange); samples cluster without obvious batch-driven separation. **(B)** Pairwise QC-QC comparisons with kernel densities and scatterplots; all QC correlations are very high (r ≈ 0.992-0.994), indicating stable instrument performance. **(C)** Post-processing boxplots of log-scaled feature intensities across all runs (QC, STS, LTS) demonstrating comparable distributions. **(D)** Unsupervised hierarchical clustering of samples further supporting absence of spurious grouping and overall platform consistency.

Supplementary Figure 2 Schoenfeld residual diagnostics for the proportional hazard’s assumption in the Cox model for overall survival. Panels display scaled Schoenfeld residuals over follow-up time for the metabolite-based risk score, tumor grade, and PD-L1 CPS score, respectively. The points represent covariate-specific residuals, and the solid curves depict smoothed trends, with the horizontal reference line indicating zero effect.

Supplementary Figure 3 Decision-curve analysis (DCA) for 12-month overall survival. Net benefit is plotted against threshold probability for 12-month mortality, comparing the metabolite-based risk score model (“RiskScore only”), the clinicopathologic model (tumor grade and PD-L1 CPS; “Clinicopathologic”), and the combined nomogram (“Combined nomogram”) with the default “Treat all” and “Treat none” strategies. Across a wide range of clinically relevant threshold probabilities, the combined nomogram yields higher net benefit than either the risk score alone, the clinicopathologic model, or the default strategies, indicating superior potential clinical utility for guiding risk-adapted management.
