# Supplementary figures and images for: Identification of plasma lipidomic biomarkers for prognostic stratification in advanced gastric cancer treated with PD-1 inhibitor plus chemotherapy
Source: Front Immunol. 2026 Feb 9;17:1714472. doi: 10.3389/fimmu.2026.1714472 (PMC12926363; doi:10.3389/fimmu.2026.1714472)

A

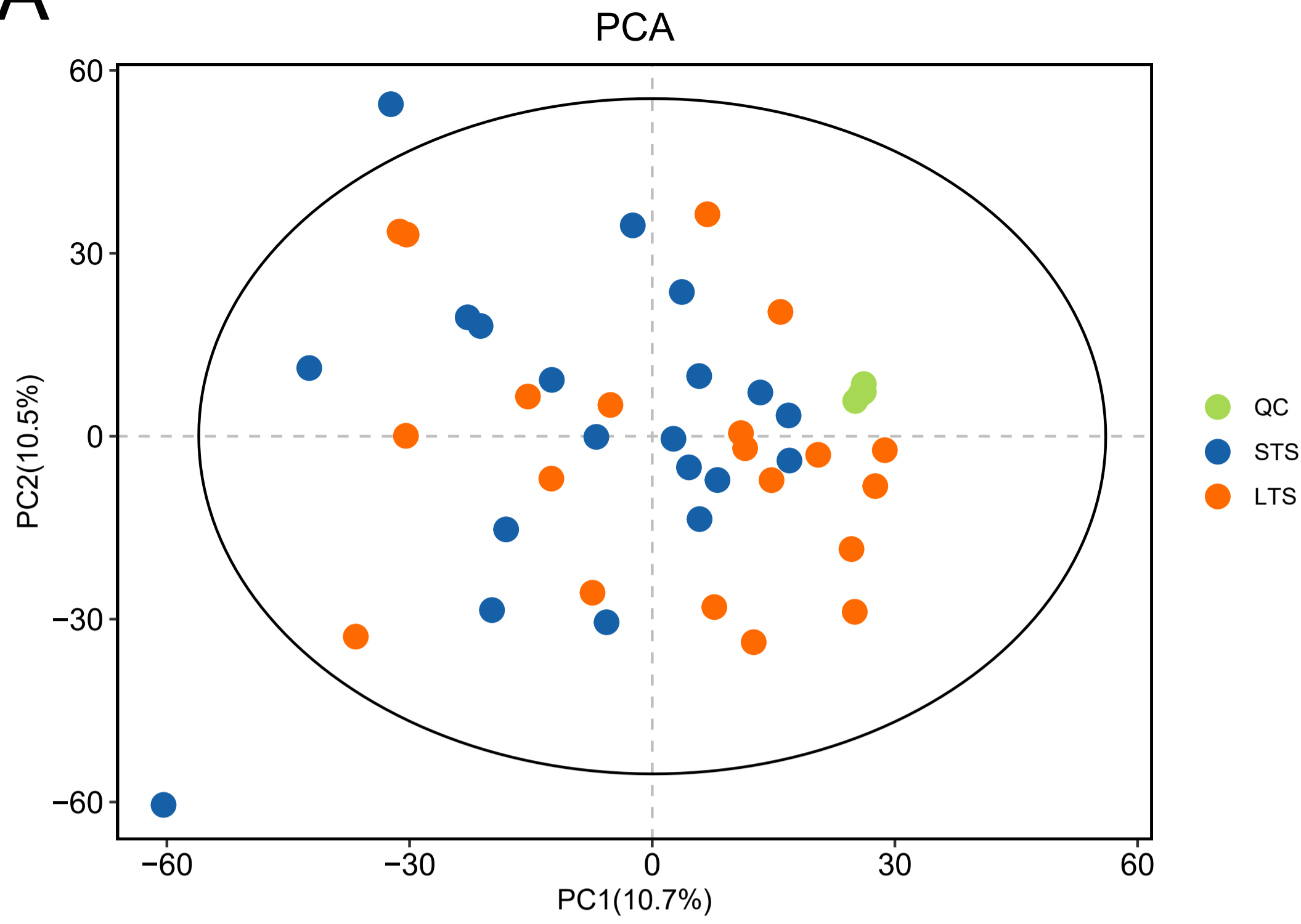

B

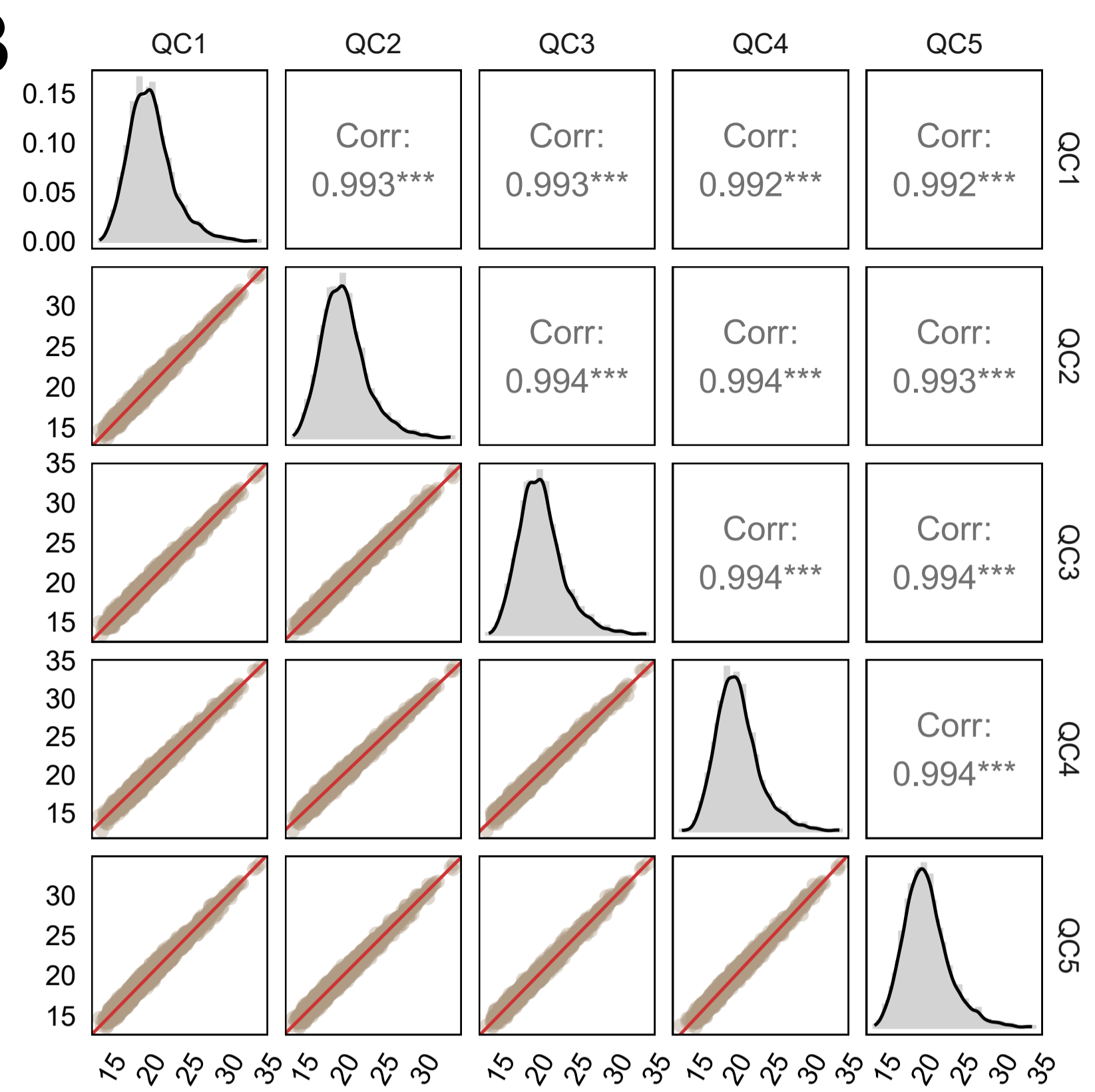

C

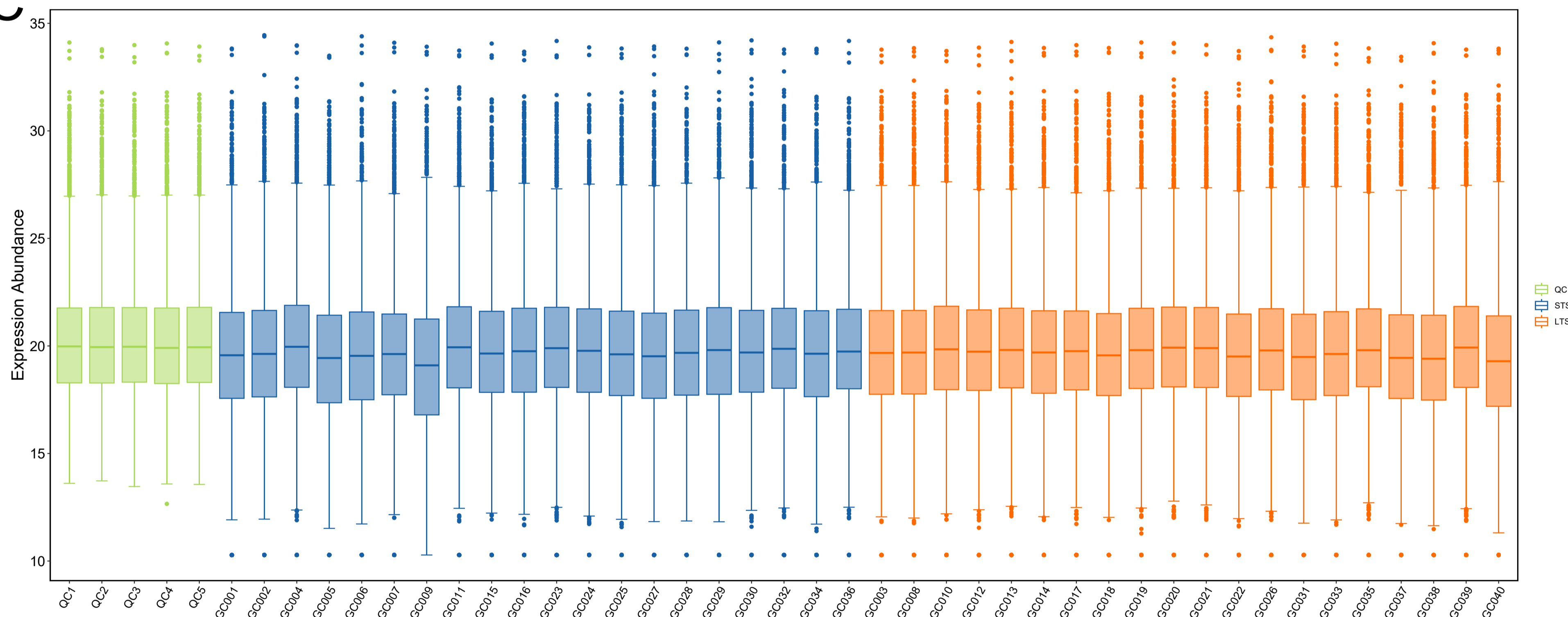

D

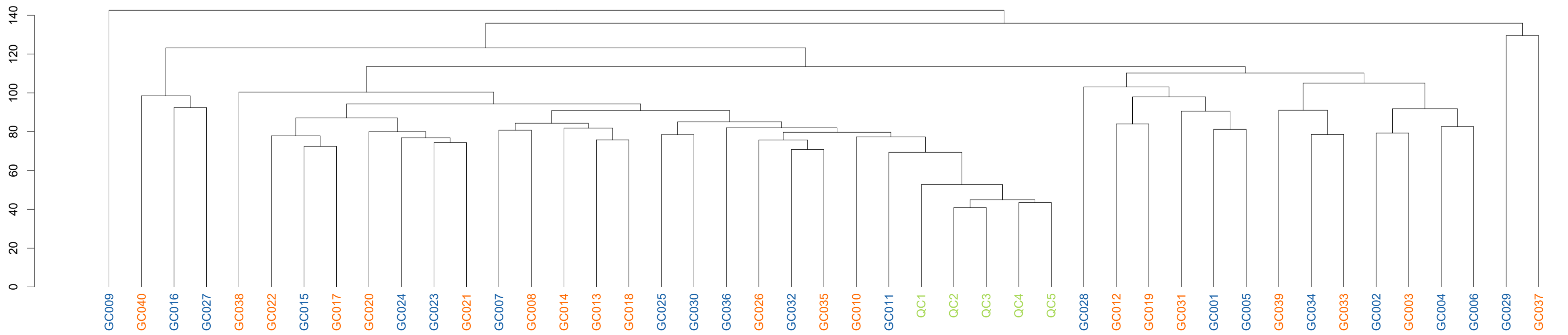

Supplement: Supplementary Figure 1 — QC assessment and global structure of the lipidomics dataset. (A) PCA score plot showing overall dispersion of pooled-QC (green), short-term survivors (STS, blue), and long-term survivors (LTS, orange); samples cluster without obvious batch-driven separation. (B) Pairwise QC-QC comparisons with kernel densities and scatterplots; all QC correlations are very high (r ≈ 0.992-0.994), indicating stable instrument performance. (C) Post-processing boxplots of log-scaled feature intensities across all runs (QC, STS, LTS) demonstrating comparable distributions. (D) Unsupervised hierarchical clustering of samples further supporting absence of spurious grouping and overall platform consistency. [file DataSheet1.pdf]

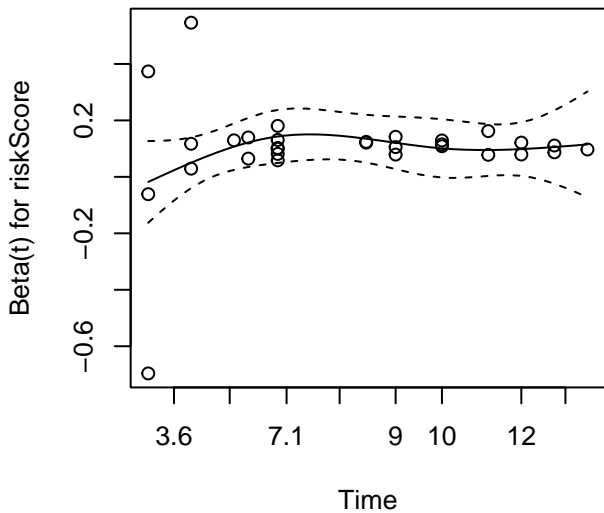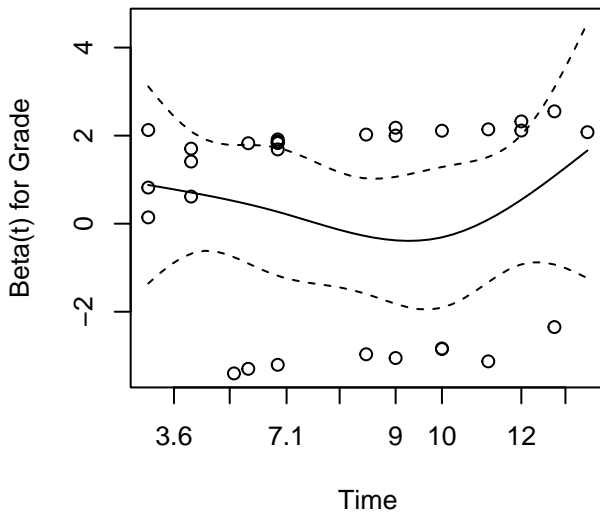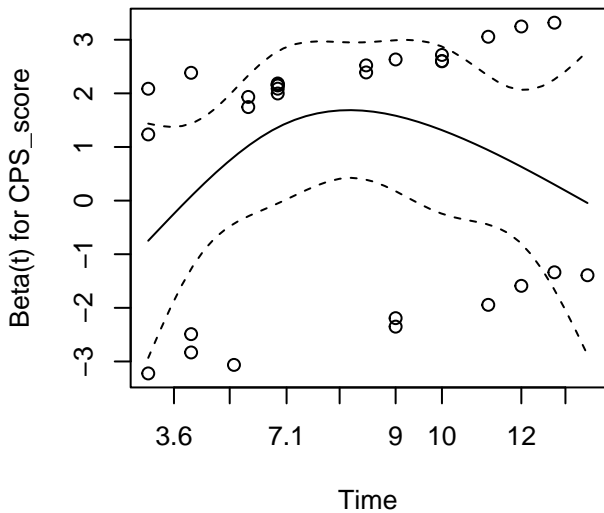

Supplement: Supplementary Figure 2 — Schoenfeld residual diagnostics for the proportional hazard’s assumption in the Cox model for overall survival. Panels display scaled Schoenfeld residuals over follow-up time for the metabolite-based risk score, tumor grade, and PD-L1 CPS score, respectively. The points represent covariate-specific residuals, and the solid curves depict smoothed trends, with the horizontal reference line indicating zero effect. [file DataSheet2.pdf]

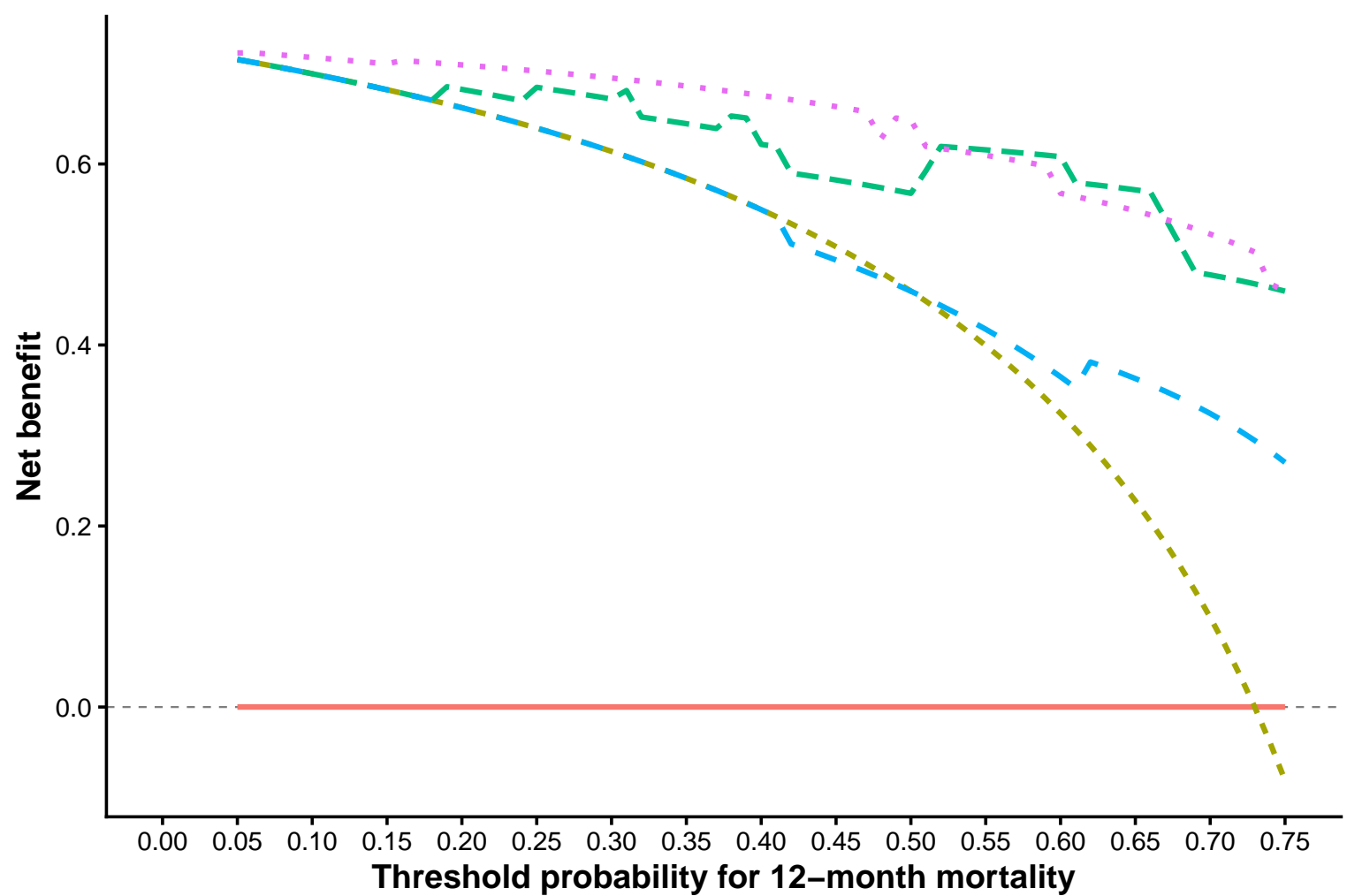

— Treat none    - - - Treat all    - - - RiskScore only    - - - Clinicopathologic    - - - Combined nomogram

Supplement: Supplementary Figure 3 — Decision-curve analysis (DCA) for 12-month overall survival. Net benefit is plotted against threshold probability for 12-month mortality, comparing the metabolite-based risk score model (“RiskScore only”), the clinicopathologic model (tumor grade and PD-L1 CPS; “Clinicopathologic”), and the combined nomogram (“Combined nomogram”) with the default “Treat all” and “Treat none” strategies. Across a wide range of clinically relevant threshold probabilities, the combined nomogram yields higher net benefit than either the risk score alone, the clinicopathologic model, or the default strategies, indicating superior potential clinical utility for guiding risk-adapted management. [file DataSheet3.pdf]
