# Supplementary material for: Identification of plasma lipidomic biomarkers for prognostic stratification in advanced gastric cancer treated with PD-1 inhibitor plus chemotherapy
Source: Front Immunol. 2026 Feb 9;17:1714472. doi: 10.3389/fimmu.2026.1714472 (PMC12926363; doi:10.3389/fimmu.2026.1714472)
Supplement: Supplementary file 4 [file Table1.docx]

Supplementary Table 1. Bootstrap performance evaluation metrics for OS prediction models at 6, 12, and 18 months.

| Performance Metric | Apparent value | Optimism-corrected value (95% CI) |
| --- | --- | --- |
| C-index | 0.7519 | 0.7292 (0.7120, 0.8070) |
| AUC_6m | 0.7142 | 0.6964 (0.6762, 0.7952) |
| AUC_12m | 0.8534 | 0.8206 (0.7889, 0.9404) |
| AUC_18m | 0.8293 | 0.7933 (0.7815, 0.9304) |
